# Calculation of the Global and Local Conceptual DFT Indices for the Prediction of the Chemical Reactivity Properties of Papuamides A–F Marine Drugs

**DOI:** 10.3390/molecules24183312

**Published:** 2019-09-11

**Authors:** Norma Flores-Holguín, Juan Frau, Daniel Glossman-Mitnik

**Affiliations:** 1 Laboratorio Virtual NANOCOSMOS, Departamento de Medio Ambiente y Energía, Centro de Investigación en Materiales Avanzados, Miguel de Cervantes 120, Complejo Industrial Chihuahua, Chihuahua 31136, Mexico; norma.flores@cimav.edu.mx; 2 Departament de Química, Universitat de les Illes Balears, 07122 Palma de Mallorca, Spain; juan.frau@uib.es

**Keywords:** Papuamides, Chemical Reactivity Theory, pKa, bioavailability, bioactivity scores

## Abstract

A well-behaved model chemistry previously validated for the study of the chemical reactivity of peptides was considered for the calculation of the molecular properties and structures of the Papuamide family of marine peptides. A methodology based on Conceptual Density Functional Theory (CDFT) was chosen for the determination of the reactivity descriptors. The molecular active sites were associated with the active regions of the molecules related to the nucleophilic and electrophilic Parr functions. Finally, the drug-likenesses and the bioactivity scores for the Papuamide peptides were predicted through a homology methodology relating them with the calculated reactivity descriptors, while other properties such as the pKas were determined following a methodology developed by our group.

## 1. Introduction

The marine environment is considered Earth’s last frontier of exploration. In fact, a common belief is that just less than 5% of the vast and rich marine environment has been explored. Our seas and oceans represent a very unknown resource for the discovery of novel organisms, (bio)products, (bio)processes, and for the development of bioinspired synthetic drugs. Recent advances in genetics and other (bio)molecular techniques are providing all necessary tools to access these still-untapped marine resources on a larger scale and, consequently, enabling exploitation of the true promise of the blue biotechnology [1].

The marine environment is acquiring even more interest as a source of new bioactive compounds, among these marine organisms derived bioactive peptides are considered a promising group of natural substances exhibiting different biological activities: antimicrobial, anticancer, antihypertensive, anti-inflammatory and so on. In particular, antimicrobial activity may counter the emerging severe problem of antibiotic-resistant microorganisms. Among marine animals, sponges have recently attracted the great interest of pharmacologists, chemists and biochemists as a rich source of peculiar antimicrobial compounds that they have evolved to protect themselves due to their sessile nature. Sponges produce a great variety of antimicrobial peptides with peculiar structural features showing antifungal, antibacterial and antiviral properties [2]. Much of contemporary investigation in the life sciences is devoted to the molecular-scale understanding of the relationships between genes and the environment, in particular dynamic alterations in the levels, modifications, and interactions of cellular effectors, including peptides and proteins [3,4].

Bioactive peptides are promising novel drug leads that may fill the gap between small molecules and larger biologicals. This is reflected by a multitude of recent peptide discovery and development approaches. However, their use as therapeutic lead molecules is challenged by their typically poor stability and lack of oral bioavailability. This is often due to the linear nature of peptides that not only exhibit free ends but also multiple cleavage sites that are readily recognized by enzymes that degrade peptide chains into inactive fragments or single amino acids [5,6,7,8,9,10,11,12,13,14].

Several cyclodepsipeptides have been identified in recent years from marine sponges with antimicrobial and antiviral activities [15,16,17,18]. These cyclic peptides have a peculiar structure, including several unusual residues and unique N-terminal polyketide-derived moieties. Papuamides A–D from sponges of *Theonella* sp. are representative cyclic depsipeptides, having cytoprotective activity against HIV in vitro, by inhibiting viral entry [19]. Similarly, some bioactive peptides demonstrate multifunctional activities based on their structure and factors such as charge, hydrophobicity, and binding properties [20,21].

Marine bioresources are a valuable source of bioactive compounds with industrial, nutraceutical and therapeutical potential [22,23,24]. Papuamides are a class of marine sponge-derived cyclic depsipeptides, which are thought to have cytoprotective activity against HIV in vitro through the inhibition of viral entry [25,26,27,28].

One of the priority approaches to discovering drug candidates is the targeting of proteases, which are pertinent drug targets in cancer, as well as cardiovascular, inflammatory, and infectious disease areas. Proteases are enzymes that play essential functions in many signaling pathways, the development of certain types of cancer, and in infectious diseases such as malaria and trypanosomiasis. The marine sponge *Theonella swinhoei* has shown to be a source of antiprotease and anti-HIV secondary metabolites. The marine sponge *Theonella affmirabilis* has been reported to contain the protease inhibitor Miraziridine A and Papuamides A and B with anti-HIV properties [29].

For the consideration of natural products as the basis for the development of of new pharmaceutical drugs, it is of paramount importance to have a comprehension of the way in those molecules interact with their potential biological receptors. From the point of view of Computational Chemistry, this can be achieved by resorting to some useful concepts that arise from Density Functional Theory (DFT), the so called Conceptual DFT [30,31,32,33,34,35,36]. The importance of using theoretical simulation such as DFT calculations in predicting physicochemical and biological characteristics of molecules and drugs has recently been highlighted [37,38,39,40]. Understanding the reactivity properties of the Papuamides family of marine peptides is important in the use of the Conceptual DFT to represent the peptides reactivity within the molecular systems in the process of developing new drugs based on them.

Indeed, other useful information as the potential drug-likeness based on the oral bioavailability, a measure of their bioactivity in the form of bioactivity scores using a homology modeling or comparison with molecules of known bioactivity, the identification of the possible sites of interaction or drug receptors and the pharmacokinetics of the studied peptides can be achieved by resorting to readily available software for searching in public databases.

As a follow up of previous research on the subject [41,42,43,44,45], this study sought to assess the chemical reactivity properties of the Papuamides family of marine peptides through the application of the Density Functional Theory concepts as well as the determination of their drug-likeness, bioactivity scores and pharmacokinetics based on the online Molinspiration program and on SwissADME, a free web tool for the evaluation of those properties [30,31,32,46,47,48].

## 2. Computational Methodology

The generation of 3D structures and the proposition of their respective low energy conformers in the prediction and calculation of the properties of the six members of the Papuamides family of marine peptides in this study were carried out using the Marvin View 17.15 program (ChemAxon, Budapest, Hungary) through the included ChemAxon Calculator plugins. Marvin View is an advanced chemical viewer that is believed to be suitable to single and multiple chemical structures, reactions and queries. The most stable conformers were chosen by performing molecular mechanics calculations with the different torsional angles being involved through the overall MMFF94 force field [49,50,51,52,53]. In the process of geometry reoptimization, the conformers with the lowest energy for each peptide were considered by using the DFTBA program (Density Functional Tight Binding Model A) available within Gaussian 09 [54]. The MN12SX/Def2TZVP/H20 model chemistry was then used in a new reoptimization of the resultant structures obtained through the DFTBA calculations. Consequently, the real minimum approach was used in the confirmation of the optimized Papuamides structures through the application of the vibrational frequency analysis technique. In the process of calculating the electronic properties for the chemical reactivity of the antimicrobial peptides involved the use of MN12SX/Def2TZVP/H2O model chemistry through the optimized molecular structures, as explained in detail in Section 3. The choice of this model chemistry was based in the fact that, according to our previous research [41,42,43,44,45], it allows obtaining HOMO (Highest Occupied Molecular Orbital) and LUMO (Lowest Unoccupied Molecular Orbital) energies that verify the KID procedure (Koopmans in DFT) making easier the determination of the chemical reactivity descriptors arising from Conceptual DFT.

## 3. Results and Discussion

**Figure 1:**
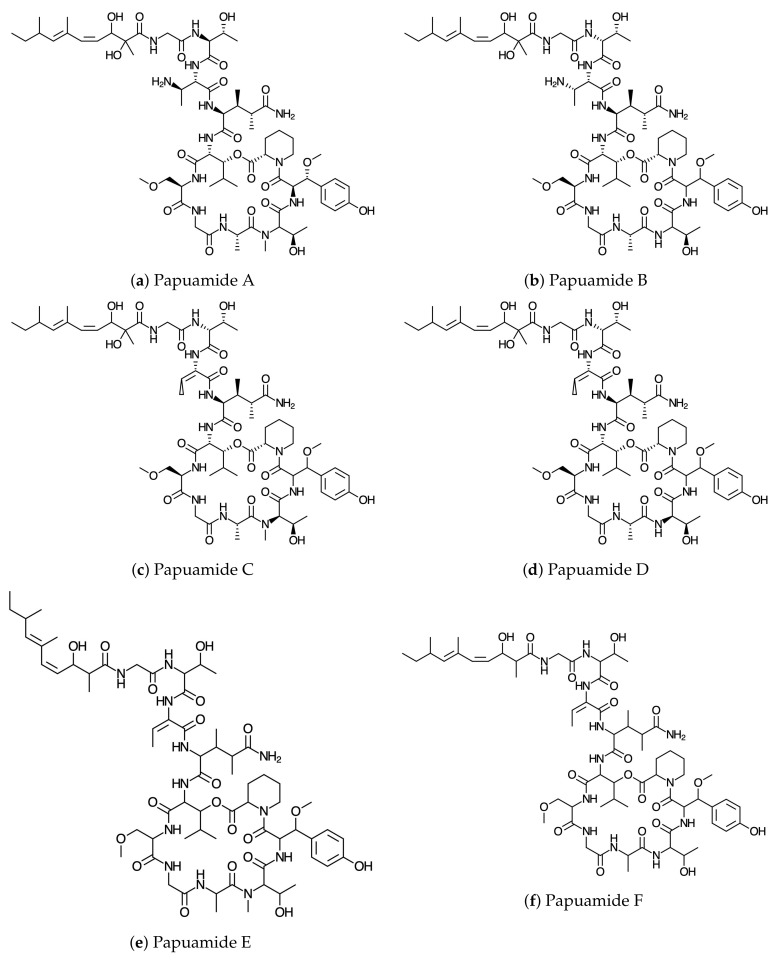
Graphical sketches of the molecular structures of: (**a**) Papuamide A; (**b**) Papuamide B; (**c**) Papuamide C; (**d**) Papuamide D; (**e**) Papuamide E; and (**f**) Papuamide F.

ChemAxon Calculator plugins were used in the process of deriving the molecular structures and the bioactivity properties of the conformers. The optimization and reoptimization of the conformers was carried out using the DFTBA program and the MN12SX/DefTZVP/H20 model chemistry, respectively, as explained in Section 2 [54]. The graphical sketches of the molecular structures of Papuamides A–F are shown in Figure 1. The DFTBA method was used in the reoptimization of the molecular structures while the MN12SX density functional method [55] combined with the SMD solvent model [56], and the DefTZVP [57,58] were used in the second optimization of the molecular structures. The MN12SX/DefTZVP/H2O model chemistry was used in determining the electronic properties of each molecular structure after using calculation analysis procedures to determine whether all the molecular structures correspond to their respective minimum energy requirements. According to Becke, a common misconception exists in the connection between the Kohn–Sham (KS) electronic ground states and excitation energies [59]. Baerends et al. stated that the level of energy excitation within a KS system is used as an effective measure of the optimization to the molecular optical gap [60]. Thus, the HOMO–LUMO gap of the KS model is used to approximate the excitation energy within the KS model based, which is a basic requirement in determining the consistency with the molecular structures [61]. Ground state calculations are used in determining the optimal maximum absorption wavelength that belongs to the marine peptides of the Papuamides family based on the chosen density functional to find the respective $\lambda_{max}$ values through the application of theoretical models to establish the HOMO–LUMO gaps. Therefore, the calculation of the maximum wavelength absorption of Papuamides A–F marine peptides involved conducting ground-state calculations with the aforementioned density functional at the same level of model chemistry and theory and determining the HOMO–LUMO gap. The corresponding results are displayed in Table 1.

### 3.1. Calculation of the Global Reactivity Descriptors of the Papuamides

According to Frau and Glossman-Mitnik, the evaluation of marine peptides and melanoidins in the generation of HOMO and LUMO energies is required in the verification of the levels of agreement with the estimated Koopmans’ theorem based on the combination of the MN12SX density functional and the Def2TZVP basis set [62,63,64,65,66,67,68]. This justifies the application of the proposed KID technique.

Considering the KID technique used on the previous studies being integrated into the finite difference approximation [62,63,64,65,66,67,68], the following expressions can be used to define the global reactivity descriptors [30,31,32,69,70]:

$$\begin{array}{cc} \mathrm{Electronegativity} & \chi =-\frac{1}{2}\left(I+A\right)\approx \frac{1}{2}\left(\epsilon_{L}+\epsilon_{H}\right)\\ \mathrm{Global}\;\mathrm{Hardness} & \eta =\left(I-A\right)\approx \left(\epsilon_{L}-\epsilon_{H}\right)\\ \mathrm{Electrophilicity} & \omega =\frac{\mu^{2}}{2\eta }=\frac{{\left(I+A\right)}^{2}}{4(I-A)}\approx \frac{{\left(\epsilon_{L}+\epsilon_{H}\right)}^{2}}{4(\epsilon_{L}-\epsilon_{H})}\\ \mathrm{Electrodonating}\;\mathrm{Power} & \omega^{-}=\frac{{\left(3I+A\right)}^{2}}{16(I-A)}\approx \frac{{\left(3\epsilon_{H}+\epsilon_{L}\right)}^{2}}{16\eta }\\ \mathrm{Electroaccepting}\;\mathrm{Power} & \omega^{+}=\frac{{\left(I+3A\right)}^{2}}{16(I-A)}\approx \frac{{\left(\epsilon_{H}+3\epsilon_{L}\right)}^{2}}{16\eta }\\ \mathrm{Net}\;\mathrm{Electrophilicity} & \Delta \omega^{\pm }=\omega^{+}-\left(-\omega^{-}\right)=\omega^{+}+\omega^{-} \end{array}$$

being $\epsilon_{H}$ and $\epsilon_{L}$ the HOMO and LUMO energies associated to each of the peptides, respectively.

The calculated values for these global reactivity descriptors using the MN12SX/Def2TZVP/H2O model chemistry and the associated HOMO and LUMO energies are displayed in Table 2.

### 3.2. Calculation of the pKa of Papuamides A–F Peptides

The above discussion focuses on the application of the conceptual DFT descriptors to evaluate the computation prediction of the pKas peptides, which established that the pKa =16.3088−0.8268×η relationship would play an important role in the initial prediction of complex peptides that are important in the manufacture of medical drugs [71]. Given the biological level of pH, the peptides under study exist as neutral molecules and are still considered to be neutral during the pKa computations [71]. The pKa relationship is also important in the optimization of the molecular structure of every conformer as well as the computation of the pKa values for all molecules given the η values shown in Table 2. The computational results of the pKa values for the Papuamides molecules are shown in Table 3 below:

The pKa values shown in Table 3 indicate that the computational methodology used is effective in the differentiation of the respective pKa values for all the peptide molecules irrespective of the significance of the difference. The pKa values of these peptides are important in the manufacture of pharmaceutical drugs by explaining the procedures used in drug delivery and their respective action mechanisms.

### 3.3. Local Reactivity Descriptors Calculation

Applying the same ideas as before, the definitions for the local reactivity descriptors are [30,31,32,34,72,73,74,75,76,77,78]:
Nucleophilic Fukui Function$f^{+}\left(r\right)=\rho_{N+1}\left(r\right)-\rho_{N}\left(r\right)$
Electrophilic Fukui Function$f^{-}\left(r\right)=\rho_{N}\left(r\right)-\rho_{N-1}\left(r\right)$
Dual DescriptorΔf(r) = ${\left(\frac{\partial \;f(r)}{\partial \;N}\right)}_{\upsilon (r)}$
Nucleophilic Parr Function$P{}^{-}\left(r\right)=\rho_{s}^{rc}\left(r\right)$
Electrophilic Parr Function$P{}^{+}\left(r\right)=\rho_{s}^{ra}\left(r\right)$

where $\rho_{N+1}\left(r\right)$, $\rho_{N}\left(r\right)$, and $\rho_{N-1}\left(r\right)$ are the electronic densities at point r for a system with N+1, *N*, and N−1 electrons, respectively; and $\rho_{s}^{rc}\left(r\right)$ and $\rho_{s}^{ra}(r$) are related to the molecular spin densities (ASD) of the radical cation or anion of a given molecule, respectively [35].

**Figure 2:**
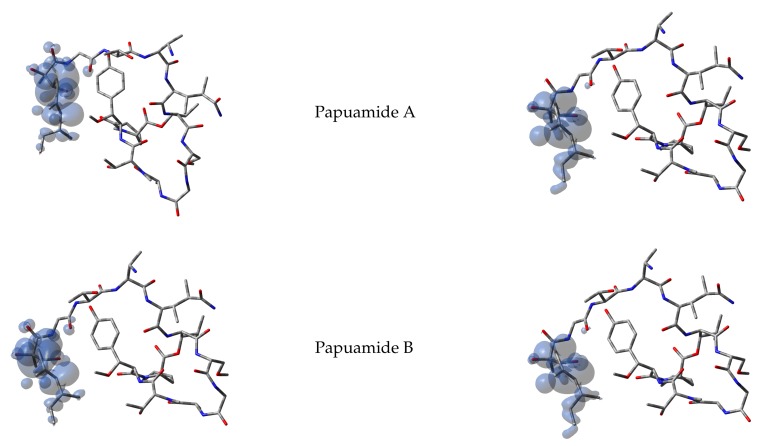

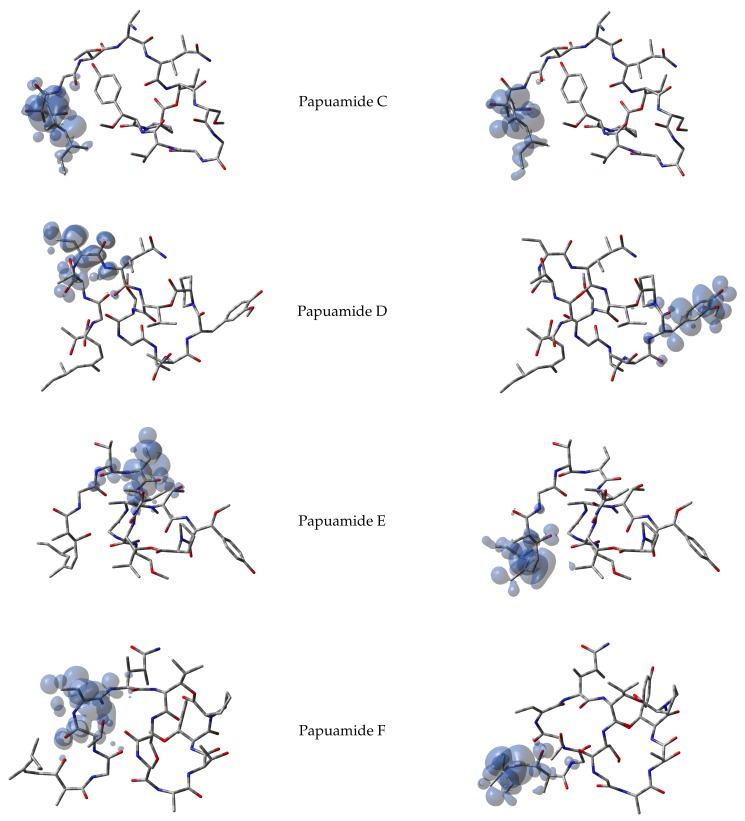
Graphical representation of the Electrophilic $P{}^{+}\left(r\right)$ (**left column**) and Nucleophilic $P{}^{-}\left(r\right)$ Parr functions (**right column**) of Papuamides A–F.

It has been argued that the Parr functions behave better than the Fukui functions in predicting the chemical reactivity of the electrophilic and nucleophilic regions of the molecules. Thus, starting from the spin densities arising from calculations for the systems with N+1 and N−1 electrons, the corresponding Parr functions for Papuamides A–F molecules are shown in Figure 2:

### 3.4. Bioavailability, Bioactivity Scores and Pharmacokinetics

According to Leeson et al, it is important to check the species level of compliance of a potential therapeutic drug to the Lipinski Rule of Five, which explains whether the compound contains drug certain drug properties [79]. Molinsipration (Slovensky Grob, Slovak Republic (www.molinspiration.com)) and MolSoft (MolSoft LLC, USA (www.molsoft.com)) software packages were used to compute the molecular drug properties in a compound by feeding the software with the corresponding SMILES (Simplified Molecular Input Line Entry Specification) notation for each peptide, as shown in Table 4, given that miLogP is a representation of the water partition coefficient. The rate of violations of the Lipinski Rule of Five is measured using nviol while TPSA represents the polar surface area of the molecule. The hydrogen bond donors and the hydrogen bond acceptors are represented by nOHNH and the nON, respectively. Volume represents the molecular volume of the peptides while MW represents the molecular weight of the peptides.

Table 4 displays the application of the Lipinski Rule of Five in the calculation of the molecular properties of the Papuamides family of marine peptides:

The degree of oral bioavailability of the marine molecules that can be potentially used in the manufacture of drugs is measured using the Lipinski Rule of Five by determining the molecules that possess drug-like properties. However, this technique could not be applied in measuring the bioavailability of the peptides due to the existence of hydrogen bonds and high molecular weight properties [80,81], as shown in Table 4 above.

This study applied a different technique in the evaluation of the chemical structure of other compounds that were predicted to possess similar pharmacological properties as the Papuamides peptides under study. As illustrated in Section 2, the evaluation of the pharmacological properties of different compounds in the process of determining the bioactivity scores can be carried out using Molinspiration software based on the variability of the drug targets, as shown in Table 5. According to the table, the organic molecules whose bioactivity score is less than zero are considered to be active, the organic molecules whose bioactivity score are between zero and negative five are considered to be moderately active, and the organic molecules with a score of less than negative five are considered to be inactive. Table 5 shows the bioactivity scores of the Papuamides family of marine peptides based on the interactions as enzyme inhibitors, GPCR ligands, as protease inhibitors or ion channel modulators, kinase inhibitors, and with nuclear receptors.

All peptides that were considered during this study were found to have moderate bioactivity scores for all the interactions. However, by resorting to the online SwissADME program [48] within the SwissTargetPrediction module and with the consideration of the mentioned SMILES notations, it was possible to understand their behavior separately. For instance, if considering Papuamide A, the main interaction will be with a member of the GPCR family (G-protein-coupled receptors), in this case GHRELIN or Growth Hormone-releasing Peptide Receptor. For the case of Papuamide B, it was found that it will behave as a protease inhibitor by interacting with Renin Angiotensinogenase. Turning our attention to Papuamide C, it will also act as a protease inhibitor, being its target the Human Leukocyte Elastase, and the same result was obtained for Papuamides D–F.

The knowledge of the pharmacokinetics of the molecules is a key factor in the development of new pharmaceutical drugs. The consideration of SwissADME [48] enabled the estimation for a chemical to be substrate of Glycoproteins (P-gp) or inhibitor of the most important Cytochromes P450 (CYP) isoenzymes as well of the Gastroinstestinal (GI) absorption properties and the Blood–Brain Barrier (BBB) permeation. The results for the Papuamides family of marine peptides are shown in Table 6:

## 4. Conclusions

Along this research, the chemical reactivity of a group of the six members of the Papuamides family of marine peptides was studied by resorting to the Conceptual DFT as a tool to explain the molecular interactions.

The information about the global and local reactivity descriptors of the marine peptides acquired in this work could be helpful to assist in the design of new pharmaceutical drugs based on these compounds.

Among the many descriptors that could be useful for the development of new medicines, the pKa is of paramount importance because it is related to the water solubility of drugs. Thus, when the experimental values of the pKa are unknown, the approximate QSAR relationship employed in this work could be a useful predictive tool for the determination of the pKas of peptides of similar size to those considered for the development of the equation.

The molecular properties related to drug-likeness based on the bioavailability have been predicted using a methodology already described in the literature and the descriptors used for the quantification of the bioactivity allowed the characterization of the studied peptides in relation with their behavior as protease inhibitors or by interaction with GPCRs with predicted receptors. By resorting to the online SwissADME program, it was possible to understand their behavior separately. When considering Papuamide A, the main interaction will be with a member of the GPCR family (G-protein-coupled receptors), in this case GHRELIN or Growth Hormone-releasing Peptide Receptor. For the case of Papuamide B, it was found that it will behave as a protease inhibitor by interacting with Renin Angiotensinogenase. Turning our attention to Papuamide C, it will also act as a protease inhibitor, being its target the Human Leukocyte Elastase, and the same result was obtained for Papuamides D–F. Finally, the pharmacokinetics of Papuamides A–F has been estimated by resorting to a free Web tool and the results can be of importance as a guide in their future consideration as new therapeutic peptides.

## Figures and Tables

**Table 1:** Electronic energies of the neutral molecular systems (in au) of Papuamides A–F, the HOMO and LUMO orbital energies as well as the HOMO–LUMO gap (in eV), and the maximum absorption wavelengths $\lambda_{max}$ (in nm) calculated with the MN12SX density functional and the Def2TZVP basis set using water as solvent simulated with the SMD parametrization of the IEF-PCM model.

Molecule	Total Electronic Energy	HOMO	LUMO	HOMO-LUMO Gap	$\lambda_{\max}$
Papuamide A	−4867.9217	−6.2330	−1.3679	4.8651	255
Papuamide B	−4828.6735	−6.2099	−0.9578	5.2521	236
Papuamide C	−4811.3616	−6.1394	−1.2773	4.8691	255
Papuamide D	−4772.1357	−6.0954	−1.4942	4.6012	269
Papuamide E	−4736.1517	−6.0698	−1.6403	4.4295	280
Papuamide F	−4696.9247	−6.0219	−1.3361	4.6858	265

**Table 2:** Global reactivity descriptors of Papuamides A–F, calculated with the MN12SX density functional with the Def2TZVP basis set and the SMD solvation model using water as the solvent.

Molecule	Electronegativity	Global Hardness	Electrophilicity
Papuamide A	3.8005	4.8651	1.4844
Papuamide B	3.5839	5.2521	1.2228
Papuamide C	3.7084	4.8621	1.4142
Papuamide D	3.7948	4.6012	1.5648
Papuamide E	3.8550	4.4295	1.6776
Papuamide F	3.6790	4.6858	1.4442
**Molecule**	**Electrodonating Power**	**Electroaccepting Power**	**Net Electrophilicity**
Papuamide A	5.1731	1.3726	6.5458
Papuamide B	4.5657	0.9819	5.5476
Papuamide C	4.9865	1.2781	6.2645
Papuamide D	5.3146	1.5199	6.8345
Papuamide E	5.5595	1.7044	7.2639
Papuamide F	5.0208	1.3419	6.3627

**Table 3:** pKas of Papuamides A–F.

Molecule	pKa
Papuamide A	12.29
Papuamide B	11.97
Papuamide C	12.29
Papuamide D	12.50
Papuamide E	12.65
Papuamide F	12.43

**Table 4:** Molecular properties of Papuamides A–F peptides calculated to verify the Lipinski Rule of Five.

Molecule	miLogP	TPSA	nAtoms	nON	NOHNH
Papuamide A	−3.73	517.53	100	34	18
Papuamide B	−3.97	526.32	99	34	19
Papuamide C	−1.86	491.51	99	33	16
Papuamide D	−2.11	500.30	98	33	17
Papuamide E	−0.97	471.98	98	32	15
Papuamide F	−1.22	480.07	97	32	16
**Molecule**	**Nviol**	**Nrotb**	**Volume**	**MW**	
Papuamide A	3	27	1309.85	1416.64	
Papuamide B	3	27	1292.91	1402.61	
Papuamide C	3	26	1292.30	1399.61	
Papuamide D	3	26	1275.35	1385.58	
Papuamide E	3	26	1284.63	1383.61	
Papuamide F	3	26	1267.68	1369.58	

**Table 5:** Bioactivity scores of Papuamides A–F calculated on the basis of GPCR Ligand, Ion Channel Modulator, Nuclear Receptor Ligand, Kinase Inhibitor, Protease Inhibitor and Enzyme Inhibitor interactions.

Molecule	GPCR Ligand	Ion Channel Modulator	Kinase Inhibitor
Papuamide A	−3.93	−3.99	−4.01
Papuamide B	−3.93	−3.98	−4.00
Papuamide C	−3.93	−3.99	−4.01
Papuamide D	−3.93	−3.99	−4.01
Papuamide E	−3.92	−3.99	−4.01
Papuamide F	−3.92	−3.99	−4.01
**Molecule**	**Nuclear Receptor Ligand**	**Protease Inhibitor**	**Enzyme Inhibitor**
Papuamide A	−4.00	−3.88	−3.94
Papuamide B	−3.99	−3.88	−3.94
Papuamide C	−4.00	−3.88	−3.94
Papuamide D	−4.00	−3.88	−3.93
Papuamide E	−4.00	−3.88	−3.94
Papuamide F	−4.00	−3.88	−3.93

**Table 6:** Pharmacokinetics of Papuamides A–F marine peptides.

Molecule	GI	BBB	P-gp	CYP1A2	CYP2C19
	Absorption	Permeant	Substrate	Inhibitor	Inhibitor
Papuamide A	Low	No	Yes	No	No
Papuamide B	Low	No	Yes	No	No
Papuamide C	Low	No	Yes	No	No
Papuamide D	Low	No	Yes	No	No
Papuamide E	Low	No	Yes	No	No
Papuamide F	Low	No	Yes	No	No
**Molecule**	**CYP2C9**	**CYP2D6**	**CYP3A4**	**Log Kp (cm/s)**	
	**inhibitor**	**inhibitor**	**inhibitor**	**(skin permeation)**	
Papuamide A	No	No	No	−15.67	
Papuamide B	No	No	No	−15.71	
Papuamide C	No	No	No	−14.62	
Papuamide D	No	No	No	−14.66	
Papuamide E	No	No	No	−13.85	
Papuamide F	No	No	No	−13.90	

## Data Availability

All the data and results from this study are available from the authors by request.
